# Oculomotor and Vestibular Findings in Gaucher Disease Type 3 and Their Correlation with Neurological Findings

**DOI:** 10.3389/fneur.2017.00711

**Published:** 2018-01-15

**Authors:** Tatiana Bremova-Ertl, Raphael Schiffmann, Marc C. Patterson, Nadia Belmatoug, Thierry Billette de Villemeur, Stanislavs Bardins, Claudia Frenzel, Věra Malinová, Silvia Naumann, Juliane Arndt, Eugen Mengel, Jörg Reinke, Ralf Strobl, Michael Strupp

**Affiliations:** ^1^German Center for Vertigo and Balance Disorders, University Hospital Munich, Munich, Germany; ^2^Graduate School of Systemic Neurosciences, Ludwig-Maximilians University of Munich, Munich, Germany; ^3^Institute of Metabolic Disease, Baylor Scott & White Research Institute, Dallas, TX, United States; ^4^Department of Neurology, Mayo Clinic Children’s Center, Rochester, MN, United States; ^5^Department of Pediatrics, Mayo Clinic Children’s Center, Rochester, MN, United States; ^6^Department of Clinical Genomics, Mayo Clinic Children’s Center, Rochester, MN, United States; ^7^Referral Center for Lysosomal Diseases, Department of Internal Medicine, University Hospital Paris Nord Val-de-Seine, Assistance Publique-Hôpitaux de Paris, Paris, France; ^8^Sorbonne Universités, UPMC, GRC ConCer-LD and AP-HP, Hôpital Trousseau, Service de Neuropédiatrie – Pathologie du développement, Centre de référence des malformations et maladies congénitales du cervelet, Paris, France; ^9^Department of Neurology, University Hospital Munich, Munich, Germany; ^10^First Faculty of Medicine, Department of Pediatrics and Adolescence Medicine, Charles University, General University Hospital Prague, Prague, Czechia; ^11^Villa Metabolica, Center for Paediatric and Adolescent Medicine, University Medical Center of the Johannes Gutenberg University, Mainz, Germany; ^12^Institute for Medical Information Processing, Biometrics and Epidemiology, Ludwig-Maximilians University of Munich, Munich, Germany

**Keywords:** Gaucher disease type 3, neuronopathic Gaucher disease, metabolic disease (inherited), neuro-ophthalmology, ocular motility, saccades, horizontal supranuclear saccade palsy, vertical supranuclear saccade palsy

## Abstract

**Objectives:**

To evaluate the function of the oculomotor and vestibular systems and to correlate these findings with the clinical status of patients with Gaucher disease type 3 (GD3). The goal of this cross-sectional and longitudinal study was to find oculomotor biomarkers for future clinical trials.

**Methods:**

Twenty-six patients with GD3 were assessed for eligibility and 21 were able to perform at least one task. Horizontal and vertical reflexive saccades, smooth pursuit, gaze-holding, optokinetic nystagmus, and horizontal vestibulo-ocular reflex (VOR) were examined by video-oculography/video-head impulse test and compared concurrently with 33 healthy controls. The Scale for the Assessment and Rating of Ataxia (SARA), the modified Severity Scoring Tool (mSST), and Grooved Pegboard Test (GPT) were administered to assess overall neurological function. Eleven patients were also re-assessed after 1 year.

**Results:**

Nine out of 17 patients exhibited gaze-holding deficits. One patient had upbeat nystagmus. Three patients presented with bilateral abducens palsy in combination with central oculomotor disorders, suggesting a bilateral involvement of the abducens nucleus. Horizontal angular VOR gain was reduced in all patients (0.66 ± 0.37) compared with controls (1.1 ± 0.11, *p* < 0.001). Most strongly correlated with clinical rating scales were peak velocity of downward saccades (SARA: ρ = −0.752, *p* < 0.0005; mSST: ρ = −0.611, *p* = 0.003; GPT: ρ = −0.649, *p* = 0.005) and duration of vertical saccades (SARA: ρ = 0.806, *p* < 0.001; mSST: ρ = 0.700, *p* < 0.0005; GPT: ρ = 0.558, *p* = 0.02) together with the VOR gain (SARA: ρ = −0.63, *p* = 0.016; mSST: ρ = −0.725, *p* = 0.003; GPT: ρ = −0.666, *p* = 0.004). Vertical smooth pursuit gain decreased significantly at follow-up.

**Interpretation:**

This study shows neuronal degeneration of the brainstem and cerebellum with combined involvement of both supranuclear and nuclear oculomotor structures and the vestibular system in GD3. We also identified oculomotor parameters that correlate with the neurological status and can be used as biomarkers in future clinical trials.

## Introduction

Gaucher disease (GD) is an autosomal recessive lysosomal storage disease caused by a deficiency of the acid β-glucosidase, leading to glucosylceramide accumulation, primarily in the liver, spleen, and bone marrow (1). Chronic neuronopathic GD (type 3, GD3) is a Gaucher subtype characterized by progressive neurological manifestations associated with glycolipid accumulation in the CNS (2, 3).

The disease has a variable clinical picture, but all GD3 patients exhibit slowing of horizontal saccades leading to complete horizontal saccadic palsy. As the disease progresses, vertical saccades also become slower (1, 4). As horizontal and later vertical saccades are highly impaired early in the course of the disease, they were used as a primary endpoint to investigate treatment with miglustat (5). A study of two siblings with GD3 suggested that the vestibulo-ocular reflex (VOR) might also be impaired in GD3 patients (6). Although saccadic eye movement abnormalities have been described, a comprehensive study of all forms of eye movements in GD3, including pursuit and vestibular-related movements in a large cohort of GD3 patients, has not been published yet.

Therefore, we performed a systematic examination of the oculomotor and vestibular function in GD3 patients, including a follow-up after 1 year, with the additional aim to find surrogate biomarkers for future clinical trials.

## Subjects and Methods

### Subjects

We conducted a prospective multicenter cohort study. Twenty-six patients from Germany, France, and the Czech Republic were screened for eligibility to participate in the study. Twenty-one patients (11 females) with genetically and clinically confirmed GD3, with a mean age of 17.89 ± 10.98 years (age ± SD), mean disease duration of 17.4 ± 12 years, and mean age at disease onset of 3.7 ± 5 years, who were able to perform at least one study task were included in this study.

Patients’ characteristics and correlations of clinical and oculomotor outcomes are listed in Table 1 and Table S1 in Supplementary Material. Eleven patients were assessed at two time points (longitudinal analysis over 12 months). In these patients, data from the last visit of patients were used (Table S1 in Supplementary Material).

**Table 1:** Patients’ characteristics.

Patient no.	Age of onset/age of diagnosis (years)	First symptom observed, age (years)	Medication	Genotype	Neurologic and psychiatric findings	Systemic manifestation and other findings	MRI findings	Ophthalmologic and clinical oculomotor findings	IQ
**PATIENTS FOLLOWED UP LONGITUDINALLY**									

1	1/1	Oculomotor disturbance (not specified), 19	Imiglucerase 3,200 U/2 weeks, eliglustat 100 mg/day, spironolactone 150 mg/day, calcium 1,500 mg/day, potassium 7,200 mg/day, magnesium 450 mg/day, vitamin D 3,000 U/day, pantoprazole 40 mg/day	*GBA*: W5333X S384F + R535C	Hyperreflexibility, pyramidal signs, clonus, stance and gait ataxia, dystonia	Fixed kyphosis	N	Alternating convergent squint operatively corrected, bilaterally, persisting esotropia R, visual acuity 0.5 R, 1.0 L, horizontal saccade paresis, slow vertical saccades down > up, impaired horizontal > vertical OKN, bilaterally impaired VOR	79

2	0/0	Retarded motor development, Oculomotor disturbance (not specified), 16	Imiglucerase 2,000 U/2 weeks	*GBA*: L444P L444P	Discrete stance and gait ataxia, dysmetria, hyperreflexibility, cognitive impairment	Hepatosplenomegaly, chronic obstructive lung disease	NP	Alternating convergent squint, visual acuity 0.8 R, 0.8 L, abducens palsy bilaterally, slow saccades horizontal > vertical, smooth pursuit impaired vertical > horizontal, horizontal impaired OKN, impaired VOR bilaterally	68

3^a^	1/1	Oculomotor disturbance (not specified), 2	Imiglucerase 1,600 U/2 weeks	*GBA*: L444P L444P	Dystonia	Discrete hepatomegaly, chronic obstructive lung disease, bronchial asthma, atopic dermatitis, lactose intolerance	N	Slow horizontal saccades, impaired horizontal OKN, discrete impaired VOR	101

4^a^	1/1	Oculomotor disturbance (not specified), 2	Imiglucerase 2,000 U/2 weeks	*GBA*: L444P L444P	Hyperreflexibility, hypotonus of lower extremities	Chronic obstructive lung disease, discrete thoracic kyphosis, hepatomegaly, neurodermatitis	N	Visual acuity 1.0 R, 1.0 L, slow horizontal saccades with curved trajectory (“around the house sign”), impaired horizontal OKN, discrete impaired VOR	105

5^b^	1/1	Oculomotor disturbance (not specified), 6	Imiglucerase 800 U/2 weeks	*GBA*: L444P D409H	N	Gibbus	NP	Visual acuity 1.0 R, 1.0 L, slow horizontal saccades, impaired horizontal OKN, impaired VOR	108

6^b^	7/7	NK	Imiglucerase 2,400 U/2 weeks	*GBA*: L444P D409H	Mild cognitive impairment	Mild chronic obstructive lung disease	Discrete unspecific white matter changes	Visual acuity 1.0 R, 0.8 L, low horizontal saccades, slow downward vertical saccades, impaired horizontal OKN, discrete impaired VOR	85

7	6/33	Oculomotor disturbance (not specified), 30 Epilepsy, 10	Imiglucerase 2,800 U/2 weeks, zonisamide 100 mg 2-0-3, citalopram 20 mg 1-0-0, clonazepam 0,5 mg 1/2-0-1, valproate 300 mg 1-0-2	*GBA*: G202R D409H	Stance and gait ataxia, epilepsy, myoclonus, spasticity, psychotic symptoms	Hepatomegaly, obstructive sleep-apnea syndrome, neurodermatitis	Unspecific white matter changes	Visual acuity 0.8 R, 0.8 L, horizontal saccade paresis L > R, slow abduction > adduction bilaterally, prolonged latency of horizontal saccades, slow vertical saccades, mild impaired smooth pursuit, impaired horizontal OKN	71

8	2/2	Retarded motor development, 29, Oculomotor disturbance (not specified)	Imiglucerase 3,200 U/2 weeks, levetiracetam 3,000 mg/day, lamotrigine 200 mg/day oxcarbazepine 2,400 mg/day	*GBA*: L444P L444P	Epilepsy, cognitive impairment, dystonia, stance and gait ataxia, pyramidal signs, clonus bilateral, hypomimia	St.p. spleen resection, femoral head necrosis, pathological right femur fracture, fixed kyphosis, restrictive lung disease	Discrete ventricle enlargement	Visual acuity 0.63 R, 0.63 L, horizontal saccade paresis, impaired horizontal OKN	68

9^c^	1/1	Retarded motor development, 16, Oculomotor disturbance (not specified)	Imiglucerase 4,400 U/2 weeks	*GBA*: L444P L444P	Stance and gait ataxia, spasticity, hyperreflexibility, dysarthria, dystonia, cognitive impairment	Fixed kyphosis	N	Slow horizontal saccades, slow downward vertical saccades, impaired horizontal and downward OKN, impaired VOR, head thrusts, operatively corrected squint, abducens palsy bilaterally R > L	60

10^c^	0/prenatal	Retarded motor development, 8, Oculomotor disturbance (not specified)	Imiglucerase 2,000 U/2 weeks	*GBA*: L444P L444P	Stance and gait ataxia, hyperreflexibility, dystonia, dysarthria, discrete cognitive impairment	Gibbus	NP	Operatively corrected squint R, esotropia R, visual acuity 0.8 R, 1.0 L, complete bilateral abducens palsy, primary position upbeat nystagmus, horizontal and vertical slow saccades, impaired horizontal smooth pursuit, impaired horizontal OKN, bilateral impaired VOR, head thrusts	60

11^b^	7/7	NK	Imiglucerase 2,000 U/2 weeks	*GBA*: L444P D409H	N	N	N	Visual acuity 1.0 R, 0.63 L, low horizontal saccades, discrete slow downward vertical saccades, impaired horizontal OKN, discrete impaired vertical smooth pursuit, discrete head thrusts	111

**PATIENTS MEASURED AT 1 TIMEPOINT**									

**Patient no./sex/age**	**Age of onset/age diagnosis**	**First symptom observed, age (years)**	**Medication**	**Genotype**	**Neurologic and psychiatric findings**	**Internal manifestation**	**MRI findings**	**Oculomotor findings (pathological)**	**IQ**

12	6 months/4	Oculomotor disturbance, 6 months	Imiglucerase 2,400 U/2 weeks	*GBA*: L444P L444P	N	Hepatosplenomegaly, chronic obstructive lung disease	N	Slow horizontal saccades with looping, impaired VOR leftward > rightward, impaired horizontal OKN	131

13	21/21	Epilepsy, 15	Imiglucerase 3,200 U/2 weeks, lamotrigine 600 mg/day, levetiracetam 1,000 mg/day, 10 gtt salbutamol, dimethindene 4 mL/day	*GBA*: N227S L424P	Epilepsy	Osteomyelofibrosis with trombocytopenia, neurodermatitis, allergic reaction on the imiglucerase therapy	N	Visual acuity 1.0 R, 1.0 L, discrete slow horizontal saccades, saccadic vertical smooth pursuit	114

14	6/10	Oculomotor disturbance, 6	Imiglucerase 3,400 U/2 weeks	*GBA*: IVS2 + 1G > A R463C	N	Hashimoto thyroiditis, hepatosplenomegaly	St. p. petrous bone cholesterol granuloma removal	Visual acuity 1.0 R, 0.63 L, slow horizontal saccades with “looping,” borderline slow vertical saccades, pathological VOR	124

15	2/4	Hepatosplenomegaly 4, Oculomotor disturbance, 8	Imiglucerase 2,800 U/kg	*GBA*: L335V L335V	Postural and extremity cerebellar ataxia, hypotonus, dyscalculia	Mild hepatosplenomegaly	Hypointensities in pallidum and thalamus	Visual acuity 0.63 R, 0.63 L, ptosis L with a Cogan-Twitch, abduction deficit L > R, abnormal smooth pursuit with “looping” upwards, horizontal slow abducting > adducting saccades, vertical slow saccades with “looping,” mild head thrusts, horizontal OKN present, but saccades impaired, impaired VOR with extremely slow compensatory eye movement, intact visual fixation-suppression of the VOR	81

16	1 month/17 months	Hepatosplenomegaly, 1 month	Velaglucerase 1,600 U/2 weeks	*GBA*: R359Q RecΔ55	N	Hepatosplenomegaly, generalized lymphadenopathy, hypotrophic habitus, trombocytopenia, neonatal ikterus, atopic eczema, ichtyosis simplex, st. p. anaphylactic reaction after the ninth dose of enzymatic therapy	NP	Visual acuity 0.7 bilaterally, impaired smooth pursuit vertically > horizontally, borderline impaired horizontal saccades	NP

17	6 months/6 months	Hepatosplenomegaly, 6 months	Imiglucerase 2,800 U/2 weeks, carbamazepine 900 mg/day, valproate 500 mg/day	*GBA*: L444P L444P	Epilepsy with a complex symptomatology, myoclonia, dyscalculia	Hepatosplenomegaly, gibbus, osteopenia, kyphoscoliosis with a thoracolumbar deformity	N	St. p. partial vitrectomy R, visual acuity 0.5 R, 0.8 L, low horizontal and downward vertical saccades, impaired vertical smooth pursuit, decreased vertical OKN, pathological VOR leftward > rightward	MoCA: 28/30

18	7/7	Oculomotor disturbance, 7 Epilepsy, 22	Imiglucerase 60 U/kg/2 weeks	*GBA*: L444PRecTL	Epilepsy, anxiety attacks, generalized hyperreflexia, discrete spasticity with positive Babinsky and slow rapid movements on the right foot, anxiety, depression	–	Medullary infiltration of the pontine, basin, femoral and tibial bones bilaterally	Convergent strabismus, anamnestic initially complete horizontal saccade palsy, at the time of examination slow horizontal saccades performable, bilateral abducens palsy, slow upward saccades, downward saccade palsy, blinking	NP

19	18/1	Diagnosis established on the basis of the diagnosis of his sister, first symptoms appeared at age 18	No therapy	*GBA*: L444PRecTL	Manic behavior, anxiety and depression	Splenomegaly	Medullary infiltration of the spine	Convergent strabismus, slow horizontal and downward saccades, pathological VOR	NP

20	3/19	NK	Imiglucerase, levetiracetam	*GBA*: L444P L444P	Epilepsy, facial dystonia, nasal speech, wide-based gait	Scoliosis, splenectomy due to spleen rupture, interstitial lung disease, abnormal bone marrow findings in MRI	NP	Convergent strabismus, pathological VOR	NP

21	12/27	Epilepsy, 12	Miglustat 300 mg/day, valproic acid 2,000 mg/day, clonazepam, bromazepam, phenobarbital 200 mg/day, zonisamide	*GBA*: L444P D409H	Becker disease, axonal neuropathy, myoclonic epilepsy, mild facial dystonia, tremor, extremity ataxia, hyperreflexia, no Babinski, no muscle weakness	Moderate hepatosplenomegaly, scoliosis, uncle and brother suffer from Becker’s muscular dystrophy	NP	Horizontal supranuclear saccade palsy with blinking and head thrusts	NP

22	1/1	Hepatosplenomegaly, 1	Imiglucerase 60 U/kg, carbamazepine, paroxetine, valium 60 U/kg, baclofen	*GBA*: L444P D409H	Myoclonic epilepsy, difficulty opening mouth, wide-based gait, axial and extremity ataxia, facial dystonia with dystonic tremor, hyperreflexia, no Babinski, high arches, hammer toes, mild lower extremity dysmetria, depression, paroxysmal aggression	Kyphosis, bone pain	Normal MRI	Convergent strabismus	NP

23	13 months/13 months	Hepatosplenomegaly, 2	Velaglucerase alfa 60 U/kg	*GBA*: D409H D409H	–	Hepatosplenomegaly, Turkish origin, two uncles of his father died of Gaucher disease	NP	Horizontal supranuclear saccade palsy with blinking	NP

24	1 month/18 months	Stridor and early psychomotor delay, 1 month	Imiglucerase	*GBA*: D409H IVS2 + 1G > A	Early psychomotor delay, walking not possible, can stand up with support, facial, cervical and acromioclavicular dystonia	Hepatosplenomegaly, stridor (diminishing under the therapy) from birth on, Cambodian origin. BAER abnormal with normal hearing	NP	Complete gaze palsy, convergent strabismus	NP

25	18 months/3 years	Early developmental delay 18 months, stance and gait ataxia, 5	Ambroxol 80 mg/day	*GBA*: R163X I260T	Myokimia, myoclonus, dystonia, startle, cloni, loss of walking ability with instability and sudden falls. Loss of speech	Nigerian origin. Hepatosplenomegaly, iron deficiency, thalassemia minor, microcytic anemia. Under therapy with imiglucerase worsening of ataxia, speech and myoclonic epilepsy with a progressive deterioration. Anamnestic reported improvement of the speech (saying words such as Maman or Au revoir), fine (playing with toys) and gross motor function (walking)	NP	Complete gaze palsy with convergent bilateral esotropia, abducens nerve palsy bilaterally	NP

26	1 month/1 year	Stridor and early developmental delay, 11 months	Ambroxol 40 mg/day	*GBA*: G377S c.141ΔAG	Developmental delay, gross motor impairment, dysphagia with growth retardation	Algerian origin. Hepatosplenomegaly, bronchial congestion with chronic stridor and cough	NP	Convergent squint	NP

*BAER, brainstem acoustic evoked responses; L, left; N, normal findings; NK, not known; NP, not performed; MRI, magnetic resonance imaging; MoCA, Montreal Cognitive Assessment; OKN, optokinetic nystagmus, R, right; VOR, vestibulo-ocular reflex*.

*^a,b,c^Indicated patients are siblings*.

Thirty-three matched healthy individuals (19 females, 18.69 ± 9.42 years) were also examined. All participants gave informed consent according to the Declaration of Helsinki. The study was approved by the local institutional review boards.

### Methods

Eye movements were recorded using a video-based eye-tracker system, with the camera mounted on the left eye [EyeSeeCam^®^ (7)] and examined as follows.

#### Saccades

Subjects made reflexive vertical and horizontal prosaccades in response to stimuli of 1.33° visual angle. Vertical saccades were elicited by stimuli of 5°, 10°, and 20° amplitude over the range ±10° from the central position. Horizontal saccades of 5°, 15°, and 30° amplitude, at a range of ±15° from the central position, were required. Participants performed seven saccades in response to the stimulus of each size along both axes. The targets were presented in pseudorandom order for 2,500 ms with additional variation of 500 ms. Patients were provided with verbal encouragement to follow the target jumps and, for patients with more advanced disease, the investigator, or administering care giver, pointed to target locations. Patients were instructed not to blink during the saccadic performance, and if they do so, then during the fixation period.

#### Smooth Pursuit

After the initial fixation period of 2 s, the target subtending visual angle of 0.57° moved in 3 cycles at 0.1 and 3 cycles at 0.2 Hz frequencies, yielding peak target velocities of 9.5°/s and 18.8°/s horizontally and 6.4°/s and 12.6°/s vertically with ±15° amplitude horizontally (right and left) from the central position, and then ±10° vertically (up and down) from the central position without a break. Smooth pursuit gain was calculated as the ratio of slow-phase eye velocity (SPV) to target velocity.

#### Gaze-Holding

Participants were asked to fixate a point of 0.57° visual angle presented on a monitor without moving their head. The point was primarily positioned at eye level to allow fixation straight ahead, then eccentricity changed from 15° left to 15° right and 10° down. The stimulus was presented for 10 s at each position. The data analysis of slow and quick phases of the gaze-holding nystagmus and drift was performed, as described previously (8–10). Frequency, peak velocity (PV), and amplitude of the 2D vector of quick phases were analyzed.

#### Optokinetic Nystagmus

The stimulus field subtended the visual angle of 28°. The stimulus consisted of alternating black-and-white stripes that moved for 15 s at a velocity of 7°/s with a white stripe of 0.67° and a black stripe of 0.29° moving to the left and to the right. Prior to each examination, a white point was presented in the primary position for 3 s. Subjects were instructed to keep gazing into the center of the pattern, to try to maintain optimal clarity of the stripes, and not to follow any one stripe deliberately.

#### Angular Horizontal VOR

Head impulses were defined based on the following criteria: 1. peak angular head velocity reached within the first 150 ms after onset of head impulse, 2. peak angular head velocity exceeded 100°/s, 3. head acceleration exceeded 1,000°/s^2^, and 4. head velocity 50 ms before onset of the impulse did not exceed 20°/s. The relevant component of head velocity cannot change sign during the impulse. Head impulses with a maximum head velocity outside 1.5-fold the interquartile range were rejected as outliers. A vector analysis of the three-dimensional input–output kinematics of the aVOR during high-acceleration rotations was expressed as gain γ (11). Participants were instructed to fixate a white point of visual angle of 0.3° positioned in the center of the monitor. 10 ± 2 head impulses were performed to each side.

#### Disease Evaluation

The overall neurological function in the GD3 patients was assessed by the modified Severity Scoring Tool (mSST) (12). Cerebellar status was evaluated by the Scale for Assessment and Rating of Ataxia (SARA) (13). Visuo-manual coordination was examined by means of the Grooved Pegboard Test (GPT). Cognition was assessed by the Wechsler Adult Intelligence Scale-Revised and the Wechsler Intelligence Scale for Children-IV (14) (WISC-IV).

### Statistical Analysis

We report mean, SD, and 95% confidence interval for continuous, absolute, and relative frequencies for categorical variables. Normality of data distribution was tested using the mean, median, SD, skewness, kurtosis, and box plots. As data were not normally distributed, related-samples Wilcoxon signed-rank test was run to determine if there were differences in measured scores between the initial examination and follow-up after 1 year. Non-paired Mann–Whitney *U*-tests were used to compare patients and healthy controls. For the group analysis, data from the follow-up visit of patients measured twice were used. For the longitudinal analysis, outcomes of 11 patients measured at two timepoints 1 year apart were compared. The two-tailed significance level was 0.05. As this is an exploratory study, no correction for multiple testing was conducted.

To assess the strength of the relationships between tested variables, Spearman’s rank correlation coefficient ρ was calculated. Patients with missing data in the outcome were excluded. Statistical analysis and figure design were performed using SPSS version 23.0.0 (IBM, New York, NY, USA).

## Results

### Ocular Motor System

**Figure 1:**
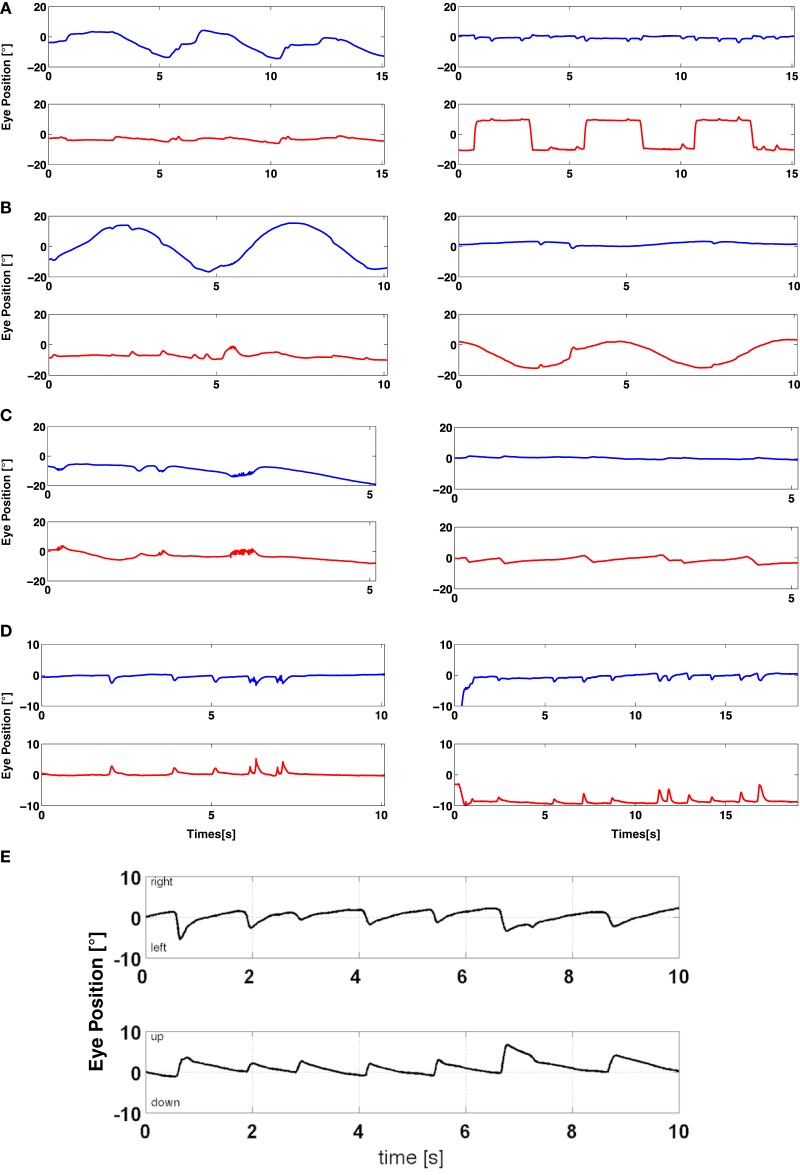
Representative raw data of oculomotor systems. A representative overview of oculomotor systems in a patient with GD3 (patient 17) and in gaze-holding in a patient with upbeat nystagmus (patient 10). *Blue lines* indicate a horizontal eye movement and *red lines* a vertical eye movement. Horizontal saccades are remarkably slow and hypometric, whereas vertical saccades are almost intact **(A)**. In contrast, horizontal smooth pursuit has a higher gain than the vertical smooth pursuit **(B)**. Horizontal optokinetic nystagmus is absent, vertical present **(C)**. The gaze-holding plot demonstrates a nystagmus-like eye movement drift, resembling saccadic pulses, which is more pronounced in the vertical plane **(D)**. Note that these pathological eye movements are present also during the saccadic and smooth pursuit examination **(A,B)**. Raw data of horizontal and vertical gaze-holding of a patient with upbeat nystagmus **(E)**. Horizontal (upper trace) and vertical (lower trace) eye movements.

Representative raw data of oculomotor systems of patient 17 are shown in Figures 1A–E.

#### Saccades

Mean asymptotic PV of both horizontal and vertical saccades in response to stimulus of 30° was 120.97°/s (83, [83.2–158.8]) (mean (±SD, [95% CIs of the mean])) in patients and 463.1°/s (77.1, [434.8–491.4]) in controls (*p* < 0.001). Mean latency was 0.269 s (0.13, [0.21–0.33]) in patients and 0.185 s (0.08, [0.157–0.213]) in controls (*p* = 0.01). Mean duration was 0.65 s (0.322, [0.5–0.79]) in patients and 0.13 s (0.04, [0.12–0.15]) in controls (*p* < 0.001). Mean gain was 0.57 (0.3, [0.44–0.71]) in patients and 0.88 (0.07, [0.85–0.9]) in the control group, respectively (*p* < 0.001). Measures of abducting vs. adducting saccades were not significantly different (Table S2 in Supplementary Material).

Mean asymptotic PV of vertical saccades in response to a stimulus of 20° was 209.7°/s (128.9, [151–268.4]) in patients and 344.5°/s (67.6, [320.9–368.1]) in controls (*p* < 0.001). Mean latency was 0.237 s (0.111, [0.187–0.287]) in GD3 and 0.198 s (0.086, [0.168–0.228]) in normal controls, respectively (*p* = 0.053). Mean duration was 0.39 s (0.34, [0.24–0.55]) in patients and 0.12 s (0.03, [0.12–0.13]) in controls (*p* < 0.001). Mean gain of vertical saccades was 0.73 (0.23, [0.63–0.83]) in patients and 0.79 (0.11, [0.75–0.83]) in controls (*p* = 0.544).

To assess overall saccadic performance compared to previous studies (4, 5), slopes of peak duration vs. amplitude were calculated by linear regression fit of the saccades. Slopes of horizontal saccades were 9.04 (10, [4.49–13.6]) in patients and 1.58 (0.5, [1.4–1.76]) in controls (*p* < 0.001). The slopes of peak duration vs. amplitude for vertical saccades were 6.9 (13, [0.98–12.8]) in patients and 1.9 (0.79, [1.6–2.2]) in the control group, respectively (*p* < 0.01). The slopes of peak duration vs. amplitude for downward vertical saccades were 10.58 (17.75, [2–19.1]) in patients and 2.19 (1.05, [1.81–2.6]) in the control group, respectively (*p* = 0.005). The slopes of peak duration vs. amplitude for upward vertical saccades were 5.43 (10.8, [0.5–10.34]) in patients and 1.71 (0.64, [1.47–1.95]) in the control group, respectively (*p* < 0.05).

**Figure 2:**
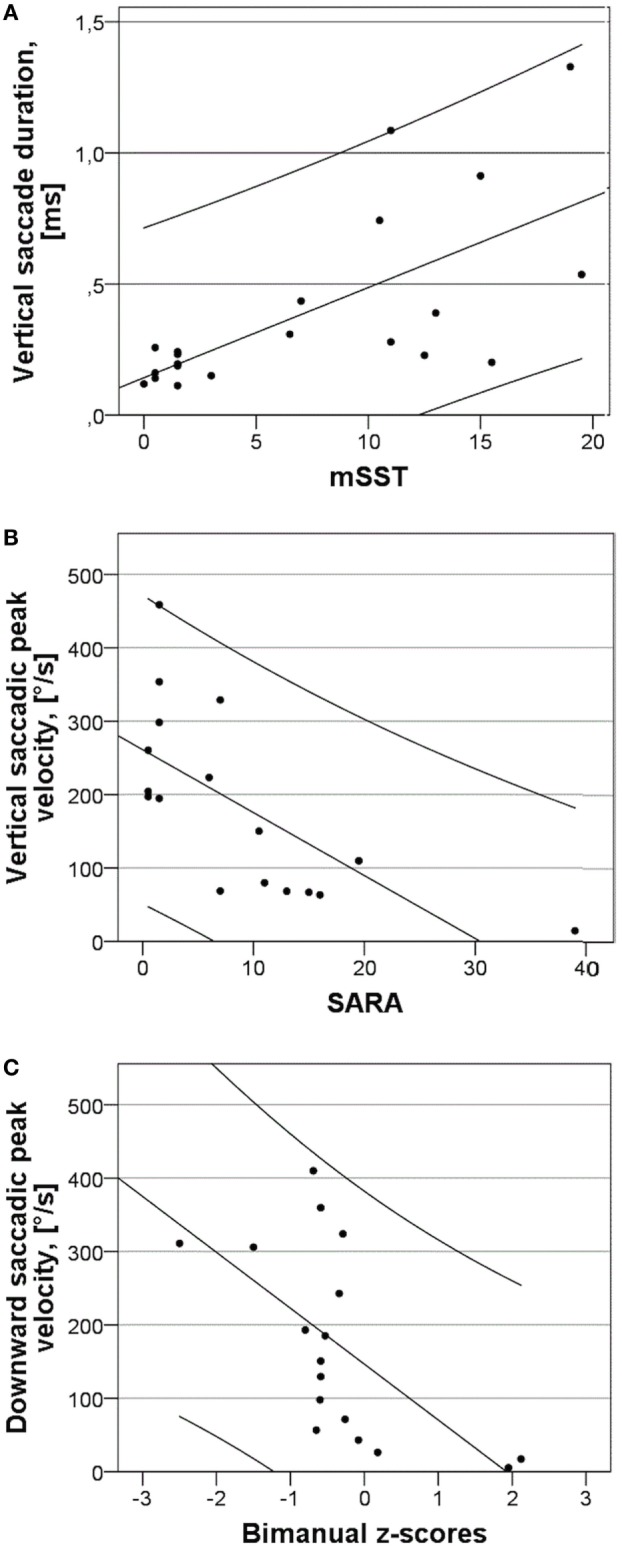
Relationships between vertical and downward saccade characteristics and clinical rating scores and slopes of saccadic peak duration vs. amplitude of horizontal and vertical saccades. **(A)** Duration of vertical saccades in response to 20° stimulus and modified Severity Scoring Tool (mSST). The line has *R*^2^ of 0.836 and the variables are significantly associated with *p* < 0.0005. **(B)** Peak velocity (PV) of vertical saccades in response to 20° stimulus and Scale for the Assessment and Rating of Ataxia (SARA). The line has R^2^ of 0.468 and the variables are significantly associated with *p* = 0.001. **(C)** PV of downward saccades in response to 20° stimuli and bimanual *z*-scores of the grooved pegboard test. The line has *R*^2^ of 0.382 and the variables are significantly associated with *p* = 0.005.

To investigate which saccadic parameters most reflect neurological status, correlation analysis between clinical rating scales and saccadic measures was performed. PV of downward saccades and duration of vertical saccades showed the strongest associations with all applied clinical parameters (Table 2; Figures 2A–C). Other measures of downward vertical saccades, such as duration, amplitude, and slopes, also showed variable but significant associations with clinical scores. This was also true for all measures of vertical saccades and all clinical measures. Slopes of horizontal and vertical upward saccades showed no significant relationships with any of the clinical measures, but the IQ score (vertical slopes) was highly correlated with all clinical rating scales (Table 2). There was no significant relationship between horizontal and vertical saccadic slopes. Age was not related to saccadic performance.

**Table 2:** Correlations of clinical rating scales, disease characteristics, and selected oculomotor parameters in Gaucher disease type 3 patients.

	Disease duration	IQ	Peak velocity of downward saccades	Duration of vertical saccades	Gaze-holding (slow-phase velocity)	Quick phases (2D vector)	Gain of upward smooth pursuit	Gain of downward smooth pursuit	Gain of horizontal smooth pursuit	Gain of horizontal aVOR	Gain of horizontal aVOR to the right	Slopes of vertical saccades	Slopes of horizontal saccades
SARA	ρ = 0.588, *p* = 0.027	ρ = −0.779, *p* = 0.001	ρ = −0.752, *p* < 0.0005	ρ = 0.806, *p* < 0.001	Vertical component in the center: ρ = −0.803, *p* = 0.001	Center frequency: ρ = 0.676, *p* = 0.008	NS	0.1 Hz	NS	ρ = −0.63, *p* = 0.016	ρ = −0.614, *p* = 0.009	NS	NS
					Vertical component down: ρ = −0.772, *p* = 0.001			ρ = 0.563, *p* = 0.036					

mSST	ρ = 0.536, *p* = 0.048	ρ = −0.804, *p* = 0.001	ρ = −0.611, *p* = 0.003	ρ = 0.700, *p* < 0.0005	Vertical component in the center: ρ = −0.804, *p* = 0.002	Center frequency: ρ = 0.688, *p* = 0.013	NS	0.1 Hz	NS	ρ = −0.725, *p* = 0.003	ρ = −0.677, *p* = 0.008	NS	NS
					Vertical component down: ρ = −0.609 *p* = 0.036			ρ = 0.538, *p* = 0.047					
					Horizontal component in the center: ρ = 0.694, *p* = 0.01								
					Horizontal component right: ρ = 0.705, *p* = 0.01								

Bimanual GPT *z*-scores (dominant vs. non-dominant hand)	NS	ρ = −0.785, *p* = 0.004	ρ = −0.649, *p* = 0.005	ρ = 0.558, *p* = 0.02	Vertical component in the center: ρ = −0.809, *p* = 0.003	NS	NS	NS	Dominant hand: 0.1 Hz: ρ = −0.627, *p* = 0.039 0.2 Hz: ρ = −0.773, *p* = 0.005	ρ = −0.666, *p* = 0.004	ρ = −0.918, *p* < 0.001	NS	NS
					Horizontal component right: ρ = 0.673, *p* = 0.023				Non-dominant hand:0.1 Hz: NS, 0.2 Hz: ρ = −0.606, *p* = 0.048				

Disease duration	–	NS	NS	NS	NS	Down: frequency	0.2 Hz	NS	NS	NS	NS	NS	NS
						ρ = 0.527, *p* = 0.03	ρ = 0.439, *p* = 0.047						

Age at onset	–	NS	NS	NS	Vertical component left: ρ = −0.555, *p* = 0.021	Center: peak velocity	0.1 Hz: ρ = 0.435, *p* = 0.049	NS	NS	NS	NS	NS	NS
					Vertical component down: ρ = −0.579, *p* = 0.015	ρ = −0.661, *p* = 0.004	0.2 Hz: ρ = 0.552, *p* = 0.009						
					Horizontal component center: ρ = −0.713, *p* = 0.001								
					Horizontal component right: ρ = −0.512, *p* = 0.036								
					Horizontal component down: ρ = −0.804, *p* < 0.001								

Slopes of downward saccades	NS	ρ = −0.720, *p* = 0.005	–	–	Vertical component in the center: ρ = −0.559, *p* = 0.03	Center: frequency	NS	NS	NS	ρ = −0.556, *p* = 0.017	ρ = −0.632, *p* = 0.005	–	NS
					Vertical component left: ρ = −0.625, *p* = 0.013	ρ = 0.511, *p* = 0.051							

Slopes of vertical saccades	NS	ρ = −0.740, *p* = 0.002	–	–	Vertical component in the center: ρ = −0.733, *p* = 0.001	Center: frequency	NS	NS	NS	ρ = −0.632, *p* = 0.005	ρ = −0.767, *p* < 0.001	–	NS
					Vertical component left: ρ = −0.663, *p* = 0.004	ρ = 0.674, *p* = 0.003							
					Horizontal component right: ρ = −0.513, *p* = 0.035								
					Vertical component down: ρ = −0.544, *p* = 0.024								

Slopes of horizontal saccades	NS	NS	NS	NS	NS	NS	NS	NS	NS	NS	NS	NS	–

Age	–	NS	NS	NS	Horizontal component straight ahead: ρ = −0.498, *p* = 0.042	NS	0.1 Hz: ρ = 0.510, *p* = 0.018	0.2 Hz: ρ = 0.466, *p* = 0.033	0.1 Hz: ρ = 0.425, *p* = 0.055;	NS	NS	NS	NS
					Horizontal component in the left position: ρ = −0.618, *p* = 0.008		0.2 Hz: ρ = 0.554, *p* = 0.009		0.2 Hz: NS				
					Horizontal component in the down position: ρ = −0.651, *p* = 0.005								

*Only significant results are shown*.

*GPT, Grooved Pegboard Test; mSST, modified Severity Scoring Tool; NS, not significant; OKN, optokinetic nystagmus; p, level of significance; ρ, Spearman correlation coefficient; VOR, vestibulo-ocular reflex; SARA, Scale for the Assessment and Rating of Ataxia*.

#### Smooth Pursuit

Since smooth pursuit matures until mid-adolescence (15, 16), children aged 4–16 and adults older than 16 were analyzed separately. This maturing process was also observed in a significant association between age and vertical smooth pursuit (upward > downward, Table 2). Table S2 in Supplementary Material provides an overview of smooth pursuit values. Downward gain at 0.1 Hz differed significantly between adult patients and controls (*p* = 0.013).

There was no difference between smooth pursuit to the left and the right, but there was a significant difference between upward and downward smooth pursuit at both frequencies in both pediatric and adult patients. Interestingly, this was also true for adult controls at 0.1 Hz. Correlation analysis showed a significant relationship between downward smooth pursuit at 0.1 Hz and mSST and SARA scores. The horizontal smooth pursuit was negatively associated with GPT *z*-scores of both hands (Table 2).

#### Gaze-Holding Function

##### Slow-Phase Velocity of Gaze-Holding Nystagmus

**Figure 3:**
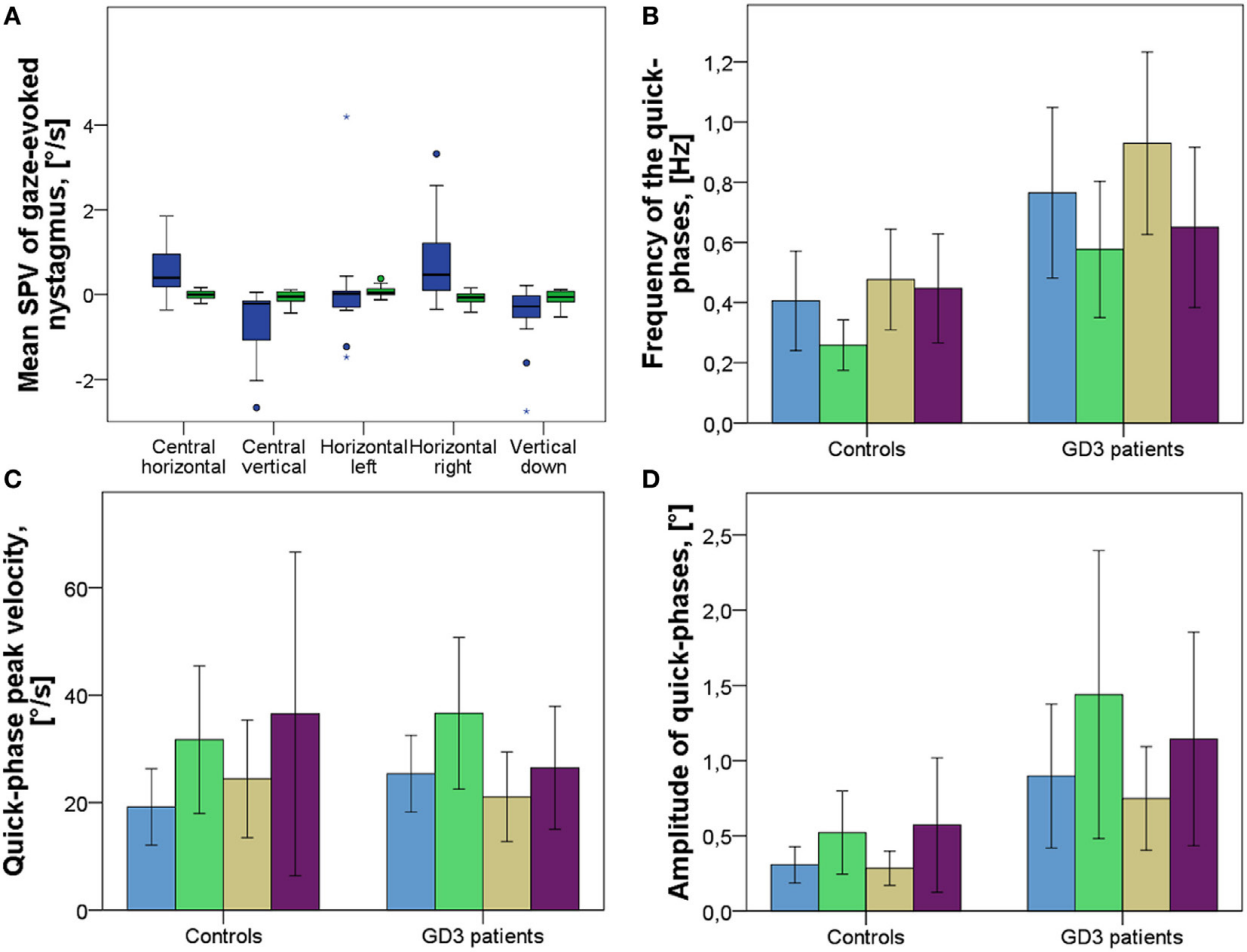
Fixation with the gaze straight ahead and gaze-holding in eccentric positions in patients and controls. **(A)** Slow-phase velocity (SPV) of eye movement drift and gaze-evoked nystagmus. *Blue* represents GD3 patients, *green* represents healthy controls. Negative values represent direction (vertical, leftwards and vertical downwards). **(B)** Frequency of the 2D vector of quick phases in the respective positions. **(C)** Peak velocity of the 2D vector of quick phases in respective positions. **(D)** Amplitude of the 2D vector of quick phases in respective positions. *Blue* represents the fixation with the gaze straight ahead. *Green* represents the gaze-holding to the left. *Khaki* represents the gaze-holding to the right. *Purple* represents the gaze-holding in the down position. The length of the boxes indicates the interquartile space (P25–P75); the horizontal line into the box represents the median (P50), and the whiskers indicate the adjacent values. The circles indicate the outliers and the stars represent an extreme value.

Nine out of 17 patients exhibited gaze-holding deficits. Patient 10 had primary-position upbeat nystagmus (Figure 1E). SPV of the horizontal eye movement drift with the gaze straight ahead differed significantly, yielding 0.48°/s (0.57 [0.19–0.77]) (mean (±SD [95% CIs of the mean])) in patients and −0.01°/s (0.09 [−0.06–0.04]) in controls (*p* = 0.001). SPV of the vertical eye movement drift in the central position was significantly different with −0.64°/s (0.74 [−0.26, −1.02]) in patients and -0.03°/s (0.1 [−0.09, −0.02]) in controls (*p* < 0.001) (Figure 3A). Gaze-holding deficits were most pronounced in patients 1, 3, 7, 17, and 21, as was SPV of the gaze-holding nystagmus in every examined position, in vertical and horizontal directions (Table S2 in Supplementary Material). In terms of correlations between neurological function and gaze-holding, vertical eye movement drift in the center and in the down position correlated significantly with all clinical tests applied, except bimanual GPT *z*-scores in the down position (Table 2). Horizontal eye movement drift in the center and right position correlated significantly with mSST and bimanual GPT *z*-scores.

##### Quick-Phase Velocity of Gaze-Holding Nystagmus and Saccadic Intrusions

During VOG recordings saccadic intrusions, such as saccadic pulses (Table S2 in Supplementary Material), were found significantly more often in patients than in controls in every position but the down position (center 0.76 (0.55 [0.48–1.05]) in GD3 and 0.5 (0.3 [0.24–0.57]) in controls *p* = 0.033; left 0.64 (0.43 [0.35–0.8]) in GD3 and 0.26 (0.16 [0.18–0.34]) in healthy controls *p* = 0.007; right 1.04 (0.54 [0.62–1.2]) in GD3 and 0.56 (0.3 [0.3–0.64]) in controls right *p* = 0.016) (mean (±SD [95% CIs of the mean])) (Figures 3B,C). This was also true for the quick-phase amplitude, expressed as a 2D vector defined by vertical and horizontal components of the eye movement (center: *p* = 0.008, left: *p* = 0.044, right: *p* = 0.011) (Figure 3D).

Quick-phase velocity differed significantly in the central position with the gaze straight ahead, but not in other positions (*p* = 0.028). Frequency of the quick phases correlated strongly with SARA and mSST, but not with bimanual GPT scores, as well as with age of onset and disease duration. It also correlated with slopes of vertical saccades (Table 2).

#### Optokinetic Nystagmus

Upward, rightward, and leftward OKN slow phases differed significantly across groups tested. Horizontal PV of rightward and leftward quick OKN phases differed significantly between patients and controls.

#### Vestibular System: Angular VOR

**Figure 4:**
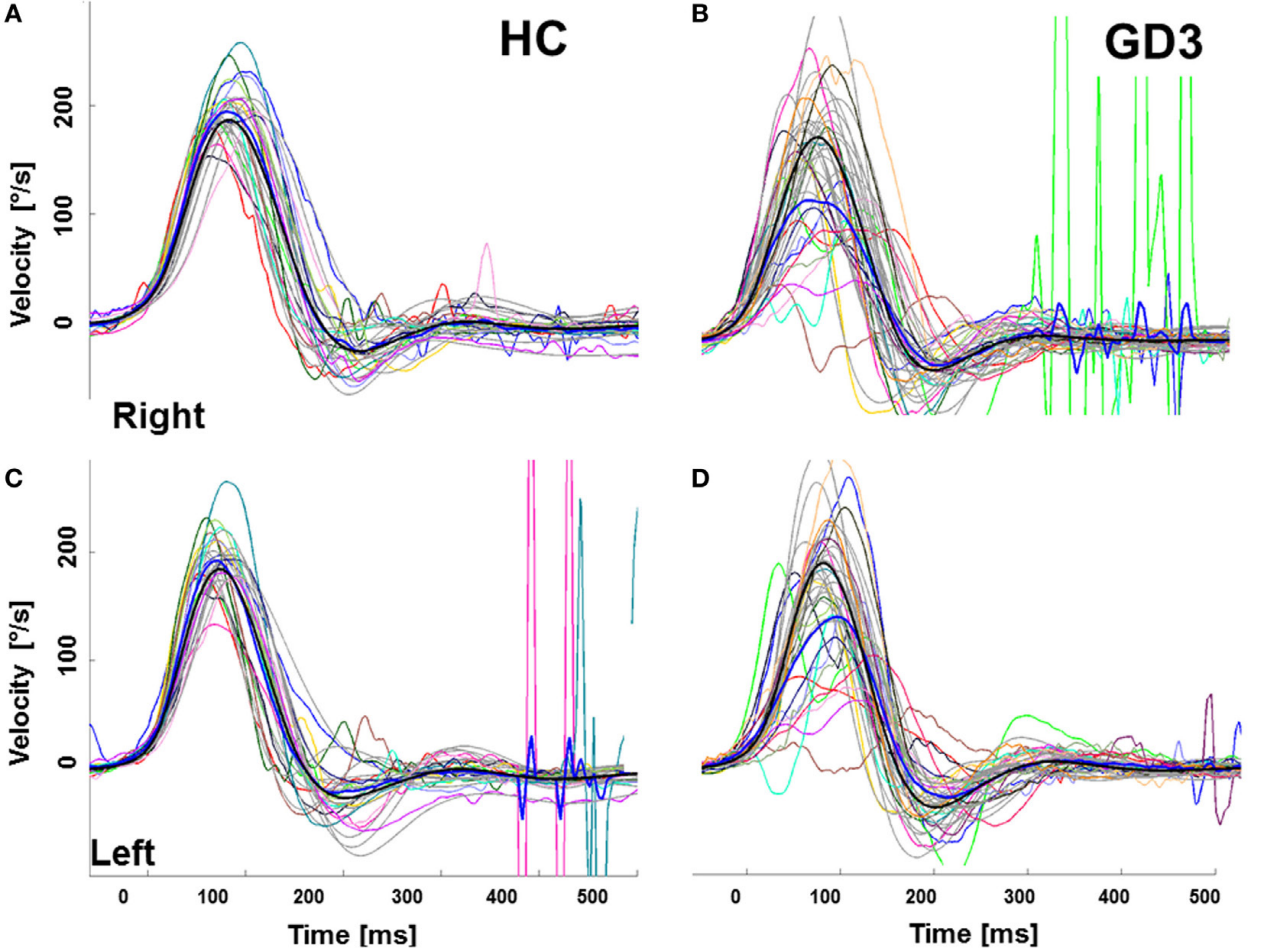
Video-head impulse test (vHIT) to assess the angular vestibulo-ocular reflex (aVOR) in healthy controls (HC) and patients with GD3. **(A,B)** vHIT to the right, **(C,D)** vHIT to the left. The gray curves represent a mean of 10 ± 5 vHIT of a particular HC and a patient, respectively. The thick *blue line* represents the groups’ mean eye movement and the thick *gray* and *black lines* represent the groups’ mean head movement. Mean gain (eye velocity/head velocity to the right and to the left) of the aVOR by 60 ms was 0.66 (0.37, [0.49–0.83]) (mean (± SD [95% CIs of the mean])) in patients and 1.1 (0.12, [1.04–1.14]) in controls (*p* < 0.001).

Mean gain (eye velocity/head velocity to the right and to the left) at 60 ms was 0.66 (0.37, [0.49–0.83]) (mean (±SD [95% CIs of the mean])) in patients and 1.1 (0.12, [1.04–1.14]) in controls (*p* < 0.001) (Figure 4; Table S2 in Supplementary Material). Correlation analysis showed strong associations between the VOR to both sides, but predominantly for rightwards head movement (eye movement leftward) and all clinical scores. VOR was also significantly correlated with the slopes of vertical saccades and downward saccades, but not with upward or horizontal saccades (Table 2).

#### Disease Characteristics

Scale for the Assessment and Rating of Ataxia and mSST were significantly associated with disease duration, but not age at onset (Table 2). Horizontal eye movement drift in all positions but left was significantly associated with age at disease onset, but not with duration. This was also true for vertical eye movement drift in the left and down position. Gain of upward smooth pursuit correlated with disease duration and age at onset. Disease duration and age at onset had no relation to horizontal smooth pursuit and horizontal aVOR.

### Longitudinal Oculomotor Analysis

**Figure 5:**
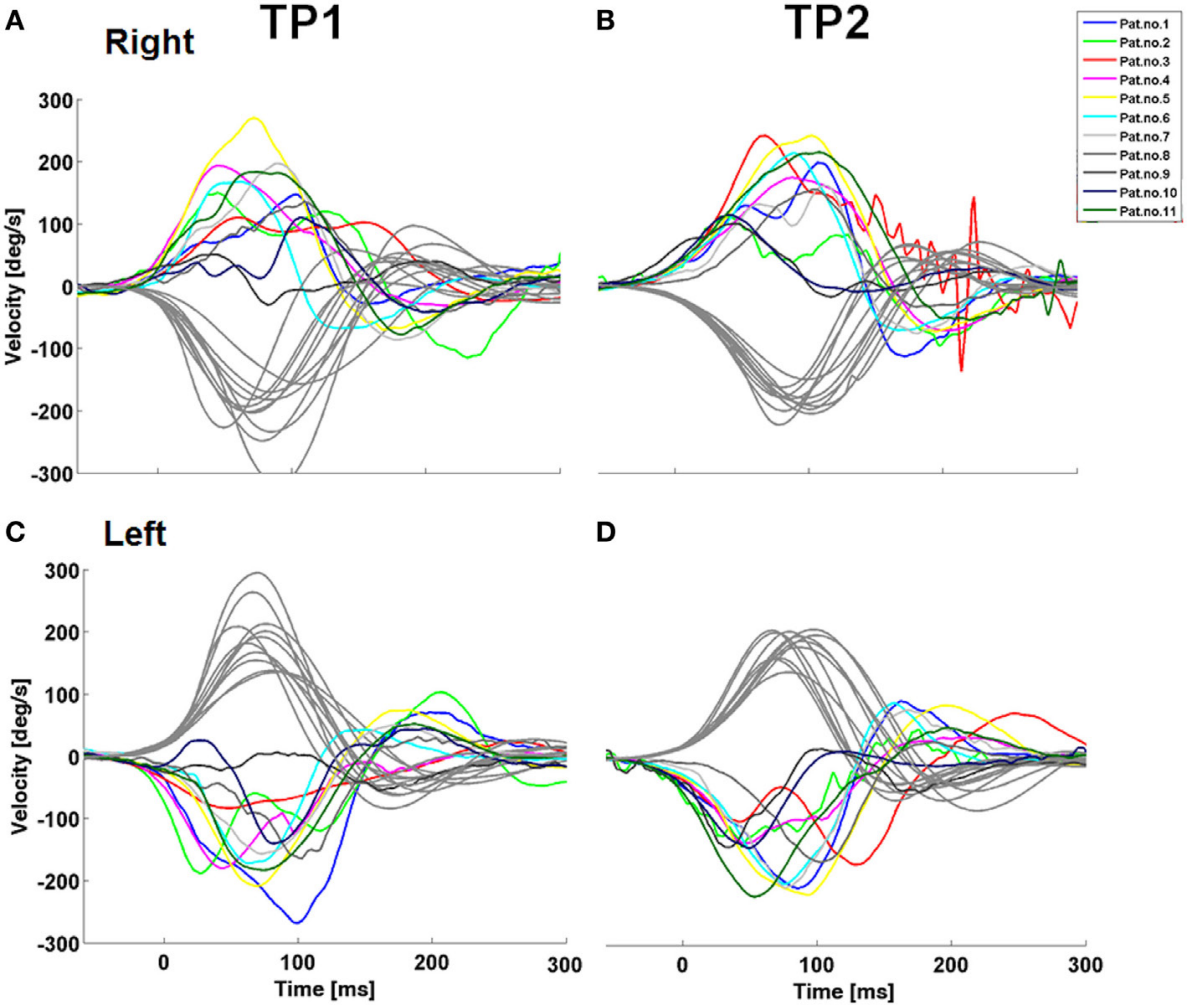
Longitudinal development of the vestibulo-ocular reflex (VOR) in patients with GD3. Note the variety of patterns of a VOR impairment which remains stable over time. The stained curves indicate the eye movement with every curve representing a mean of 10 ± 5 HIT of a particular patient. Grey depicts the head movement.

Vertical upward and downward smooth pursuit gains at 0.1 Hz, but not 0.2 Hz decreased significantly over 1 year. No other significant changes in clinical neurological scales or in saccades were observed (Table S2 in Supplementary Material). VOR to the right showed a trend to improvement after one year (Table S2 in Supplementary Material). VOR profiles were consistent over time (Figure 5).

No significant longitudinal changes in disease progression, as assessed by the clinical rating scales SARA, mSST, and GPT *z*-scores for visuo-manual dextricity, were found. Regarding individual progression, patients 2, 7, 9, and 10 worsened and patients 1 and 8 improved; the rest remained stable.

## Discussion

This study evaluated the comprehensive ocular motor and vestibular function of a sizable international cohort of patients with GD3. It related this function to their overall clinical state in a systematic and standardized manner.

The major findings in this study are as follows:

First, gaze-holding function was compromised in some patients, which might indicate a cerebellar dysfunction, mainly of the flocculus, and/or dysfunction of the neuronal integrator (17).
Second, PV of downward saccades and duration of vertical saccades as well as gains in horizontal aVOR correlated significantly with the patients’ clinical status assessed by SARA, mSST, and the GPT. Therefore, these easily quantifiable oculomotor and vestibular parameters may be used as biomarkers for future studies.
Third, horizontal aVOR was impaired in all patients and showed consistent patterns over time in patients evaluated longitudinally.
Fourth, longitudinal analysis did not show significant changes over time, except for a deterioration of vertical smooth pursuit.

Topo-anatomical findings suggest that multiple areas in the brainstem and cerebellum are involved in patients with GD3: pontine paramedian reticular formation (PPRF), rostral interstitial nucleus of the medial longitudinal fascicle (riMLF), motoneurons of the abducens nucleus, cerebellum (mainly the flocculus), and the vestibular system. For typical clinical ocular motor findings in patients with GD3, see Video S1 in Supplementary Material of patient 9 on the webpage.

### Saccades (Clinical-Anatomical Association)

Peak velocity and duration of vertical and downward saccades were the most sensitive and robust oculomotor parameters because they were strongly associated with all applied clinical rating scores. They reflect the function of the burst neurons in riMLF, since the instantaneous firing rate of these neurons closely correlates with instantaneous eye velocity (18). Slopes of linear regression fit for vertical and downward saccades also correlated with the neurological involvement; however, this parameter was not related to all neurological assessments and it had a lower significance compared to the abovementioned parameters. Interestingly, slopes of horizontal and upward saccades, calculated as peak duration (amplitude/PV) vs. amplitude were not significantly associated with any of the clinical measures, perhaps because of both a ceiling level of horizontal saccades reflecting advanced disease and upward saccades being relatively spared by the disease process. The latter is probably caused by calretinin-positive input from premotor centers, interstitial nucleus of Cajal (INC), riMLF, and y-group in upgaze, but not in downgaze pathways (19–21). Calretinin is a calcium-binding protein, and in combination with parvalbumin and perineuronal nets can help identify and analyze the upgaze vs. downgaze ocular motor disturbances in other lysosomal diseases, such as Niemann–Pick disease type C (22).

Moreover, because the riMLF innervates the upgaze motoneurons bilaterally, but downgaze motoneurons ipsilaterally, the latter are more susceptible to functional disturbances (23). Vertical, especially downward, saccades mirrored gaze-holding and aVOR, systems which also degenerate during the disease. However, in contrast to horizontal saccades, these systems do not seem to trigger a ceiling effect and therefore might potentially serve as biomarkers of disease progression. The minor vertical saccade impairment went hand-in-hand with the gaze-holding deficits in the vertical plane. The most spared upward saccades resulted in the presence of upbeat quick phases. Horizontal saccadic deficits result in an absence of all horizontal quick phases (including optokinetic and vestibular ones), as was shown in a prior publication (6). Our study confirms this finding, even though there was still a horizontal component to the movement. This might be explained by a cross-coupling mechanism between the vertical and horizontal saccadic systems (24).

Bilateral abduction deficits suggest an impairment of abducens motoneurons. Thus, patients suffer more pronounced impairment of the abducens nucleus than of the PPRF with burst neurons for horizontal saccades, which leads to an isolated saccadic impairment. The common feature of these patients is bilateral esotropia, which is further accentuated by convergence. Notably, these patients were all homozygous for the L444P mutation.

The prolonged latency of horizontal saccades indicates an impairment of initiation of reflexive saccades that can be also related to the PPRF disturbance. Since GD3 patients exhibit deficits of reflexive saccades, quick phases, and vestibular movements *per se*, head thrusts do not represent oculomotor apraxia, defined as voluntary eye movement failure, but probably represent an attempted adaptive strategy to facilitate change in gaze, as you can see in Video S2 in Supplementary Material (25). We observed the same variation of saccadic PV in the phase plane plots of eye position vs. velocity as seen in a previous study (4). This is due to the malfunction of omnipause neurons in the pontine nucleus raphe interpositus that control saccade generation by inhibiting the activity of burst neurons (26).

### Vestibulo-Ocular Reflex

We have observed similar velocity “wiggling” during the performance of VOR, with each patient having a unique VOR pattern, as you can see in the Figure 5. A trajectory analysis revealed substantial decelerations of eye velocity, which appeared variably during the VOR course. A pathological VOR gain has been previously described in 2 siblings with GD3 (6). The pathological VOR is probably caused by a complex involvement of abducens nuclei, the medial vestibular region and/or vestibulo-cerebellum, accounting for reduced VOR gains, and by the PPRF, responsible for an absence of re-fixation saccades. Moreover, since some patients were not able to counteract the head movement at all, they generated a delayed saccadic movement to the fixation target; thus, the disturbance of the omnipause neurons might also explain transient decelerations during the VOR course.

### Smooth Pursuit

In terms of smooth pursuit, only downward gain differed between adult patients and controls, being better in patients. This might be explained by riMLF and/or cerebellar impairment, leading to diminished saccadic intrusions during the smooth pursuit. Smooth pursuit seemed to be relatively spared by the disease process, although downward and upward smooth pursuit both significantly worsened at follow-up. Also, downward gain correlated significantly with the mSST and SARA scores. Therefore, downward gain might be a suitable biomarker, despite its age-dependence. The low values of the vertical smooth pursuit gain are based on the fact that orientation in the horizontal plane is more important than in the vertical one for humans as bipedal animals. This was also reflected in the negative association between horizontal smooth pursuit and the GPT, since both saccades and smooth pursuit are needed for the performance of complex tasks in daily life.

### Gaze-Holding

Gaze-holding deficits were found in fixation both with gaze straight ahead and gaze-holding in eccentric positions, suggesting that neural integrators and the vestibulo-cerebellum might be involved (see also Figure 3). The gaze-holding nystagmus had a predominant vertical upbeat component, with a minor horizontal component, seen also in correlations with all clinical rating scores. This reinforces the possibility of cross-coupling mechanism, as inactivation of PPRF also leads to saccades with curved trajectories in monkeys (27).

The eye motility deficits seen in some patients might be gaze-paretic in nature, related to an abnormal innervation pulse to the lateral recti muscles, which are not able to abduct the eyes due to the abducens dysfunction. Eleven patients were diagnosed with convergent squint and in three cases strabismus was surgically corrected. Mild amblyopia was seen in 10 out of 11 patients with strabismus. The convergent squint might be caused by an impairment of visual fusional mechanism; however, it might be related to the impairment of the cerebellar flocculus and, in the association with dysmetric saccades, also of the oculomotor vermis (28).

Esotropia might contribute to the fixation deficits seen in our patients, since they are well known in strabismic individuals, especially in those with amblyopia (29–32). The primary-position upbeat nystagmus is probably related to the paramedian medulla (33) (especially in light of the impaired VOR) involvement. Nevertheless, vertical eye movements in terms of vertical gaze-holding nystagmus might also be generated to compensate for the horizontal eye movement palsy due to impairment of abducens nucleus. Oculomotor systems correlated variably with disease duration and/or age of onset, reflecting a variable rate of involvement of neural structures during the course of disease.

### Longitudinal Analysis

We did not see any disease progression in the saccade analysis as a group, but the data varied. This also accounts for the lack of correlation between vertical and horizontal saccadic systems. Angular VOR to the right showed a tendency to improvement at follow-up; the VOR to the left remained quite stable. Moreover, patients showed unique patterns with variable velocity changes in the aVOR course. In contrast to saccades, downward and upward smooth pursuit worsened over 1 year. This demonstrates that in GD3 oculomotor deficits progress to different extents and at different rates.

### Limitations of the Study

The sample size for the longitudinal analysis is small, with a short follow-up length due to the logistical peculiarities and disease rarity, meaning that these analyses might be underpowered. Also, the further vestibular tests, such as vertical semicircular canal evaluation and vestibular evoked myogenic potentials (VEMP) examination, testing the otolith function were not performed, mainly due to the logistical reasons. As some patients were children and/or became fatigued very quickly, some tests were not performed due to lack of compliance or physical disability. The measurement was monocular, so that movement differences between eyes were not assessed.

## Conclusion

In conclusion, our findings suggest a widespread neuronal dysfunction, both at brainstem and cerebellar levels. The deficits seen in the oculomotor and vestibular examination, particularly those that progressed over time, can be used as biomarkers in future clinical trials. Future studies evaluating the voluntary saccades, including also absolute number of performed saccades or intersaccadic intervals, should be planned to assess the reliability of these biomarkers. The otolith function examined by VEMP holds promise as another vestibular biomarker, as was shown previously. Also, a compact binocular testing battery to minimize the effect of fatigue should be performed in clinical trials in the future. Our clinical experience also suggests performing the VOG as early as possible prior to the other clinical tests.

## Ethics Statement

This study was carried out in accordance with the recommendations of Good Clinical Practice with written informed consent from all subjects. All subjects gave written informed consent in accordance with the Declaration of Helsinki. The protocol was approved by the institutional review board of the Ludwig-Maximilians University.

## Author Note

Statistical analysis was conducted by Tatiana Bremova-Ertl, MD, PhD, University Hospital Munich and Ralf Strobl, Dr. Dipl.-Stat, Institute for Medical Information Processing, Biometrics and Epidemiology.

## Author Contributions

TB-E: design of the study, acquisition and analysis of data, figure design, and writing the manuscript. RS: conception of the study, and revising the manuscript for important intellectual content. SB: figure design and revising the manuscript for important intellectual content. MP, NB, TV, VM, SN, JA, EM, and JR: acquisition of data and revising the manuscript for important intellectual content. RS and CF: analysis of data and revising the manuscript for important intellectual content. MS: drafting and revising the manuscript for important intellectual content.

## Conflict of Interest Statement

TB-E received speaker’s honoraria from Actelion and Sanofi-Genzyme. RS received research support and honoraria from Protalix Biotherapeutics, Sanofi Genzyme, Amicus Therapeutics, and Shire. SB received speaker’s honoraria from Actelion and owns stock in EyeSeeTec GmbH. VM received speaker’s honoraria from Actelion, Sanofi-Genzyme, Shire, and Synageva. EM received speaker’s honoraria and consultant fees from Actelion, Genzyme, BioMarin, Shire HGT, Orphazyme, and Alexion. JR received speaker’s honoraria from BioMarin, Shire, Genzyme, and Actelion. MS is Joint Editor-in-Chief of the *Journal of Neurology*, Editor-in-Chief of *Frontiers of Neuro-otology*, and Section Editor of *F1000*. He received speaker’s honoraria from Abbott, UCB, GSK, TEVA, Biogen, IntraBio, Pierre-Fabre, Eisai, Sensorion, and HennigPharma. MP has received honoraria from Actelion, Amicus, Orphazyme, Shire, Stem Cells, Vtesse for service on advisory boards, research funding from Actelion, and travel funds from Actelion, Orphazyme, and Vtesse. He serves as Editor-in-Chief of the *Journal of Child Neurology* and *Child Neurology Open*, as an Editor of *Journal of Inherited Metabolic Disease* and *JIMD Reports*, and as Section Editor for Pediatric Neurology for *Up-To-Date*. NB has received speaker’s honoraria, and honoraria for service on advisory boards, research funding and travel funds from Genzyme/Sanofi and Shire. JA, SN, RS, CF, and TBV report no disclosures.

## Supplementary Material

Video S1 Thorough ocular motor examination of a patient 9.

Video S2 Impaired voluntary saccades in a patient 1, compensated by head thrusts.

## Abbreviations

CI, confidence interval; INC, interstitial nucleus of Cajal; HC, healthy controls; GD3, Gaucher disease type 3; GPT, Grooved Pegboard Test; mSST, modified severity scoring tool; OKN, optokinetic nystagmus; PPRF, paramedian pontine reticular formation; PV, peak velocity; riMLF, rostral interstitial nucleus of the medial longitudinal fascicle; SPV, slow-phase velocity; QPV, quick-phase velocity; VOR, vestibulo-ocular reflex; SARA, scale for the assessment and rating of ataxia; vHIT, video-head impulse test.
