# Supplementary material for: Oculomotor and Vestibular Findings in Gaucher Disease Type 3 and Their Correlation with Neurological Findings
Source: Front Neurol. 2018 Jan 15;8:711. doi: 10.3389/fneur.2017.00711 (PMC5775219; doi:10.3389/fneur.2017.00711)
Supplement: Supplementary file 3 [file table_1.docx]

**Supplemental table S1**

Overview of clinical rating scores in Gaucher disease type 3 patients.

**A. Patients measured longitudinally**

| **Patient number** | **Age** | **MSST1** | **MSST2** | **SARA1** | **SARA2** | **Dominant hand** | **GPT 1**  **(right/left**  **hand) [s]** | **GPT 2 (right/left hand) [s]** | **GPT normal sex- and age-matched values (right/left)^1^ [s]** | **Confidence intervals in adults and SD in children, [s] for dominant/non-dominant hand^1^** | |
| --- | --- | --- | --- | --- | --- | --- | --- | --- | --- | --- | --- |
| 1 | 19 | 13 | 13 | 8 | 7 | R | 79/69 | 56.3/77.2 | 83/83 | [37/113]/ [46/125] | |
| 2 | 10 | 7 | 7 | 9 | 10 | R | 91/174 | 166.4/174.5 | 83/90 | 36.5/28.9 | |
| 3 | 5 | 1.5 | 1.5 | 1 | 1 | R | 75.7/129 | 43.9/61.9 | 70/75 | 33.9/38.1 | |
| 4 | 5 | 1.5 | 1.5 | 0 | 0 | R | 122.8/58.9 | 56.7/59 | 66/73 | 32.3/36.8 | |
| 5 | 6 | 0.5 | 0.5 | 0 | 0 | R | 76.5/98 | 55.4/59.4 | 63/65 | 31.2/30.1 | |
| 6 | 20 | 1.5 | 1.5 | 2 | 2 | R | 76/100 | 76.5/80.2 | 83/85 | [38/117]/ [45/127] | |
| 7 | 43 | 19 | 19 | 36 | 39 | R | 300.00/269 | 300/270.5 | 92/106 | [47/134]/ [48/160] | |
| 8 | 30 | 19.5 | 19.5 | 10 | 9 | R | 106.1/106.5 | 95.6/106.6 | 88/96 | [45/137]/ [48/146] | |
| 9 | 17 | 12 | 15 | 16 | 18 | R | 122.3/146 | 131.5/143 | 82/82 | [35/107]/ [47/122] | |
| 10 | 8 | 10 | 11 | 12 | 16 | R | 241/300 | 300/300 | 38/47 | 10.4/26.8 | |
| 11 | 19 | 1.5 | 1.5 | 0 | 0 | R | 78/76 | 59.4/65.3 | 84/83 | [25/134]/[52/109] | |
| 12 | 22 | 3 | 3 | 0 | 0 | R | 74/77 | 57.6/72 | 84/87 | [40/125]/[45/131] | |
| 13 | 22 | 0.5 | 0.5 | 0 | 0 | R | 64/81 | 63.9/70.4 | 85/85 | [26/143]/[48/121] | |
| 14 | 25 | 0.5 | 0.5 | 0 | 0 | R | 59.7/59.9 | NP | 84/88 | [30/142]/[47/129] | |
| **B. Patients measured once**  **Patient**  **number Age MSST SARA Dominant hand GPT(right/left hand)** | | | | | | | |  |  |  |  |
|  |  |  |  |  |  |  |  |  |  |  |  |
| 15 | 8 | 11 |  | 33 |  | R | 116/157.8 |  | 38/41 | 9.02/14.6 |  |
| 16 | 6 | 0 |  | NP |  |  | NP |  |  |  |  |
| 17 | 25 | 15.5 |  | 6 |  | R | NP |  |  |  |  |
| 18 | 30 | 6.5 |  | NP |  | R | Grooved bimanual  Z = -1.5 |  |  |  |  |
| 19 | 33 | 1.5 |  | NP |  | R | Grooved bimanual  Z < -2.5 |  |  |  |  |
| 20 | 26 | 10.5 |  | 6 |  | R | NP |  |  |  |  |
| 21 | 37 | 12.5 |  | NP |  | R | NP |  |  |  |  |
| 22 | 48 | 14.5 |  | 13 |  | R | NP |  |  |  |  |
| 23 | 3 | 4.5 |  | NP |  | NK | NcP |  |  |  |  |
| 24 | 2 | 4 |  | NP |  | NK | NcP |  |  |  |  |
| 25 | 6.5 | 12 |  | NP |  | R | NcP |  |  |  |  |
| 26 | 1 | 14.5 |  | NP |  | R | NcP |  |  |  |  |

^1^ As indicated in the Lafayette Grooved Pegboard Test User Instructions, *Model 32025*
