# Supplementary material for: Oculomotor and Vestibular Findings in Gaucher Disease Type 3 and Their Correlation with Neurological Findings
Source: Front Neurol. 2018 Jan 15;8:711. doi: 10.3389/fneur.2017.00711 (PMC5775219; doi:10.3389/fneur.2017.00711)
Supplement: Supplementary file 4 [file table_2.docx]

**Supplemental table S2**

Summary of oculomotor parameters in all patients and in patients examined longitudinally at two timepoints compared with normal controls.

| **Parameter** | **Patient Total** | |  | **Controls** | | |  | | | | | ***Z-value*** | | ***P*-value** | | | | **1. timepoint** | | **2. timepoint** | | | ***Z-value*** | | | ***P*-value** |
| --- | --- | --- | --- | --- | --- | --- | --- | --- | --- | --- | --- | --- | --- | --- | --- | --- | --- | --- | --- | --- | --- | --- | --- | --- | --- | --- |
| ***Horizontal saccades*** | | | | | | | | | | | | | | | | | | | | | | | | | | |
| **Asymptotic peak velocity[°/s]** | 120.97±83 | |  | 463.1±77.1 | | |  | | | | | -6.07 | | <0.001** | | | | 74.8± 40.5 | | 95.2±  57.1 | | | -0.646 | | | 0.518 |
| **Latency [s]** | 0.269±0.13 | |  | 0.185±0.08 | | |  | | | | | -2.565 | | 0.01* | | | | 0.326± 0.148 | | 0.308± 0.145 | | | -0.418 | | | 0.676 |
| **Duration [s]** | 0.65±0.322 | |  | 0.13±0.04 | | |  | | | | | -6.015 | | <0.001** | | | | 0.759± 0.458 | | 0.734± 0.304 | | | -0.494 | | | 0.621 |
| **Gain** | 0.57±0.3 | |  | 0.88±0.07 | | |  | | | | | -4.075 | | <0.001** | | | | 0.388± 0.244 | | 0.47± 0.288 | | | -0.798 | | | 0.425 |
| **Slope Peak Duration vs. Amplitude** | 9.04±10  95% CI (4.49-13.6) | |  | 1.58±0.5  95% CI (1.4-1.76) | | |  | | | | | -5.229 | | <0.001** | | | | 11.1± 5.9  95% CI (6.6-15.7) | | 9± 7.65  95% CI (2.6-14.4) | | | -0.956 | | | 0.339 |
| ***Horizontal Saccades to the right (30°)*** | | | | | | | | | | | | | | | | | | | | | | | | | | |
| **Asymptotic peak velocity[°/s]** | 124.6±92.2 | |  | 466.1±87.6 | | |  | | | | | -6.014 | | <0.001** | | | | 71.6± 48.8 | | 102 ±74 | | | -0.874 | | | 0.382 |
| **Latency [s]** | 0.29±0.17 | |  | 0.197±0.1 | | |  | | | | | -2.155 | | <0.05* | | | | 0.39±0.25 | | 0.34± 0.195 | | | -0.418 | | | 0.676 |
| **Duration [s]** | 0.605±0.33 | |  | 0.134±0.04 | | |  | | | | | -5.847 | | <0.001** | | | | 0.75± 0.44 | | 0.72± 0.36 | | | -0.114 | | | 0.909 |
| **Gain** | 0.55±0.31 | |  | 0.88±0.1 | | |  | | | | | -3,982 | | <0.001** | | | | 0.37±0.24 | | 0.44±0.27 | | | -0.494 | | | 0.621 |
| ***Horizontal saccades to the left (30°)*** | | | | | | | | | | | | | | | | | | | | | | | | | | |
| **Asymptotic peak velocity[°/s]** | 117.4±79.5 | |  | 460.1±75.3 | | |  | | | | | -6.07 | | <0.001** | | | | 71.8± 41.1 | | 88.4±  45.2 | | | -0.915 | | | 0.360 |
| **Latency [s]** | 0.248±0.1 | |  | 0.173±0.07 | | |  | | | | | -2.733 | | 0.006* | | | | 0.29±0.13 | | 0.27± 0.1 | | | -0.423 | | | 0.673 |
| **Duration [s]** | 0.683±0.49 | |  | 0.128±0.06 | | |  | | | | | -5.847 | | <0.001** | | | | 0.78±0.495 | | 0.74± 0.49 | | | -0.599 | | | 0.549 |
| **Gain** | 0.59±0.3 | |  | 0.88±0.08 | | |  | | | | | -3,739 | | <0.001** | | | | 0.38±0.25 | | 0.5±0.33 | | | -0.986 | | | 0.324 |
| ***Vertical saccades*** | | | | | | | | | | | | | | | | | | | | | | | | | | |
| **Asymptotic peak velocity [°/s]** | 209.7±128.9 | |  | 344.5±67.6 | | | | |  | | | -4.002 | | <0.001** | | | | 174.1± 109.5 | | 172.2±143.2 | | | -0.624 | | | 0.533 |
| **Latency [s]** | 0.24±0.11 | |  | 0.198±0.07 | | | | |  | | | -1.932 | | 0.053 | | | | 0.28±0.13 | | 0.23±0.097 | | | -0.624 | | | 0.533 |
| **Duration [s]** | 0.39±0.34 | |  | 0.12±0.03 | | | | |  | | | -5.388 | | <0.001** | | | | 0.43± 0.21 | | 0.53± 0.4 | | | -0.098 | | | 0.922 |
| **Gain** | 0.73±0.23 | |  | 0.79±0.11 | | | | |  | | | -0.606 | | 0.544 | | | | 0.73± 0.25 | | 0.66± 0.22 | | | -0.689 | | | 0.491 |
| **Slope** | 6.9±13  95% CI (0.98-12.8) | |  | 1.9±0.79  95% CI (1.6-2.2) | | | | |  | | | -2.944 | | <0.01* | | | | 3.9± 4.7  95% CI (0.25-7.52) | | 5.5±4.1  95% CI (2.04-8.96) | | | -0.851 | | | 0.395 |
| **Parameter** | **Patient Total** | |  | **Controls** | | | | |  | | | ***Z-value*** | | ***P*-value** | | | | **1. timepoint** | | **2. timepoint** | | | ***Z-value*** | | | ***P*-value** |
| ***Upward vertical saccades in response to stimulus of 20°*** | | | | | | | | | | | | | | | | | | | | | | | | | | |
| **Asymptotic peak velocity [°/s]** | 241.1±146.5 | |  | 357.4±86.8 | | |  | | | | | -3.385 | | 0.001** | | | | 220.5± 125.1 | | 211.4±154.9 | | | -0.361 | | | 0.718 |
| **Latency [s]** | 0.240±0.122 | |  | 0.185±0.08 | | |  | | | | | -1.884 | | 0.06 | | | | 0.270±0.138 | | 0.244±0.139 | | | -0.591 | | | 0.554 |
| **Duration [s]** | 0.261±0.189 | |  | 0.115±0.05 | | |  | | | | | -4.841 | | < 0.001** | | | | 0.279±0.143 | | 0.3±0.245 | | | -0.361 | | | 0.718 |
| **Gain** | 0.734±0.229 | |  | 0.78±0.13 | | |  | | | | | -0.159 | | 0.874 | | | | 0.767±0.281 | | 0.659±0.229 | | | -1.215 | | | 0.224 |
| **Slope PD vs. Amplitude** | 5.43±10.8  95% CI (0.5-10.34) | |  | 1.71±0.64  95% CI(1.47-1.95) | | |  | | | | | -2.321 | | 0.02* | | | | 2.8±1.64  95% CI (1.5-4) | | 4.13±2.85  95% CI (1.74-6.5) | | | -0.966 | | | 0.334 |
| ***Downward vertical saccades in response to stimulus of 20°*** | | | | | | | | | | | | | | | | | | | | | | | | | | |
| **Asymptotic peak velocity [°/s]** | 178.3±127.1 | |  | 334±69.6 | | |  | | | | -4.168 | | | < 0.001** | | | | 127.7±99.4 | | 133±138.1 | | | -0.295 | | | 0.768 |
| **Latency [s]** | 0.234±0.114 | |  | 0.199±0.109 | | |  | | | | -2.164 | | | 0.03* | | | | 0.28±0.13 | | 0.22 (0.1) | | | -0.985 | | | 0.324 |
| **Duration [s]** | 0.524±0.56 | |  | 0.117±0.021 | | |  | | | | -5.223 | | | < 0.001** | | | | 0.58±0.45 | | 0.75 (0.64) | | | -0.755 | | | 0.450 |
| **Gain** | 0.722± 0.221 | |  | 0.82±0.12 | | |  | | | | -1.352 | | | 0.176 | | | | 0.7±0.25 | | 0.66 (0.22) | | | -0.492 | | | 0.622 |
| **Slope** | 10.58± 17.75  95% CI (2-19.1) | |  | 2.19±1.05  95% CI (1.8-2.6) | | |  | | | | -3.009 | | | 0.003* | | | | 4.4±28.6  95% CI (-17-26.4) | | 10.87 (10.71)  95% CI -1.92-19.8) | | | -0.105 | | | 0.917 |
| **Parameter** | **Patient Total** | |  | **Controls** | | |  | | | | ***Z-value*** | | | ***P*-value** | | | | **1. timepoint** | | **2. timepoint** | | | ***Z-value*** | | | ***P*-value** |
| ***Smooth pursuit^a^***  ***Age 4-16*** | **n = 8** | |  | **n = 7** | | |  | | | |  | | |  | | | |  | |  | | |  | | |  |
| **Gain Right 0.1** | 0.56±0.16 | |  | 0.66±0.16 | | |  | | | | -1.143 | | | 0.253 | | | | 0.83±0.33 | | 0.86±0.23 | | | -0.662 | | | 0.508 |
| **Gain Right 0.2** | 0.48±0.2 | |  | 0.66±0.2 | | |  | | | | -1.571 | | | 0.116 | | | | 0.7±0.32 | | 0.61±0.27 | | | -0.486 | | | 0.627 |
| **Gain Left 0.1** | 0.48±0.27 | |  | 0.59±0.19 | | |  | | | | -0.571 | | | 0.568 | | | | 0.75±0.38 | | 0.55±0.31 | | | -0.927 | | | 0.354 |
| **Gain Left 0.2** | 0.46±0.25 | |  | 0.63±0.26 | | |  | | | | -1 | | | 0.317 | | | | 0.63±0.37 | | 0.55±0.31 | | | -0.353 | | | 0.724 |
| **Gain Up 0.1** | 0.24±0.2 | |  | 0.3±0.09 | | |  | | | | -1.143 | | | 0.253 | | | | 0.47±0.35 | | 0.3±0.27 | | | -2.194 | | | 0.028* |
| **Gain Up 0.2** | 0.19±0.1 | |  | 0.31±0.19 | | |  | | | | -1.571 | | | 0.116 | | | | 0.43±0.35 | | 0.39±0.2 | | | -0.178 | | | 0.859 |
| **Gain Down 0.1** | 0.6±0.5 | |  | 0.37±0.23 | | |  | | | | -1.216 | | | 0.224 | | | | 0.9±0.32 | | 0.7±0.12 | | | -2.073 | | | 0.038* |
| **Gain Down 0.2** | 0.42±0.44 | |  | 0.34±0.25 | | |  | | | | -0.143 | | | 0.886 | | | | 0.72±0.28 | | 0.55±0.17 | | | -1.718 | | | 0.086 |
| **Horizontal Gain 0.1** | 0.52±0.17 | |  | 0.62±0.17 | | |  | | | | -1.143 | | | 0.253 | | | | 0.79±0.34 | | 0.7±0.22 | | | -0.397 | | | 0.691 |
| **Horizontal Gain 0.2** | 0.47±0.23 | |  | 0.64±0.24 | | |  | | | | -1.286 | | | 0.199 | | | | 0.67±0.34 | | 0.58±0.28 | | | -0.574 | | | 0.566 |
| **Vertical Gain 0.1** | 0.42±0.21 | |  | 0.34±0.14 | | |  | | | | -0.715 | | | 0.474 | | | | 0.67±0.22 | | 0.51±0.11 | | | -1.836 | | | 0.066 |
| **Vertical Gain 0.2** | 0.3±0.22 | |  | 0.34±0.19 | | |  | | | | -0.286 | | | 0.775 | | | | 0.57±0.26 | | 0.48±0.14 | | | -1.244 | | | 0.214 |
| ***Parameter*** | ***Patient Total*** | |  | ***Controls*** | | |  | | | | ***Z-value*** | | | ***P-value*** | | | | ***1. timepoint*** | | ***2. timepoint*** | | | ***Z-value*** | | | ***P-value*** |
| ***Smooth pursuit***  ***Age >16*** | **n = 13** | |  | **n = 21** | | |  | | | |  | | |  | | | |  | |  | | |  | | |  |
| **Gain Right 0.1** | 0.89±0.2 | |  | 0.84±0.09 | | |  | | | | -0.850 | | | 0.395 | | | |  | |  | | |  | | |  |
| **Gain Right 0.2** | 0.81±0.23 | |  | 0.83±0.06 | | |  | | | | -0.225 | | | 0.822 | | | |  | |  | | |  | | |  |
| **Gain Left 0.1** | 0.85±0.2 | |  | 0.82±0.1 | | |  | | | | -0.241 | | | 0.810 | | | |  | |  | | |  | | |  |
| **Gain Left 0.2** | 0.78±0.27 | |  | 0.81±0.09 | | |  | | | | -0.305 | | | 0.760 | | | |  | |  | | |  | | |  |
| **Gain Up 0.1** | 0.56±0.26 | |  | 0.58±0.19 | | |  | | | | -0.321 | | | 0.748 | | | |  | |  | | |  | | |  |
| **Gain Up 0.2** | 0.6±0.29 | |  | 0.61±0.19 | | |  | | | | -0.497 | | | 0.619 | | | |  | |  | | |  | | |  |
| **Gain Down 0.1** | 0.85±0.17 | |  | 0.71±0.13 | | |  | | | | -2.471 | | | 0.013* | | | |  | |  | | |  | | |  |
| **Gain Down 0.2** | 0.75±0.17 | |  | 0.66±0.14 | | |  | | | | -1.316 | | | 0.188 | | | |  | |  | | |  | | |  |
| **Horizontal Gain 0.1** | 0.87±0.22 | |  | 0.83±0.1 | | |  | | | | -0.321 | | | 0.748 | | | |  | |  | | |  | | |  |
| **HorizontalGain 0.2** | 0.79±0.25 | |  | 0.82±0.1 | | |  | | | | -0.257 | | | 0.797 | | | |  | |  | | |  | | |  |
| **Vertical Gain 0.1** | 0.69±0.2 | |  | 0.65±0.15 | | |  | | | | -0.818 | | | 0.413 | | | |  | |  | | |  | | |  |
| **Vertical Gain 0.2** | 0.67±0.2 | |  | 0.63±0.16 | | |  | | | | -0.578 | | | 0.564 | | | |  | |  | | |  | | |  |
| **Gaze-holding**  **(Slow-phase velocity)^b, c, d^**  **Patients Controls *Z-value horizontal P-value horizontal Z-value vertical P-value vertical*** | | | | | | | | | | | | | | | | | | | | | | | | | | |
|  | **Horizontal** | | **Vertical** | | | **Horizontal** | | | **Vertical** |  | | |  | | | | | |  | | |  | | |  |  |
| **Center** | 0.48±0.57 | | -0.64±0.74 | | | -0.01±0.09 | | | -0.03±0.1 |  | | | -3.358 | | | | | | 0.001** | | | -3.772 | | | <0.001** |  |
| **Right** | 0.63±0.81 | | -0.46±0.9 | | | -0.05±0.1 | | | -0.14±0.12 |  | | | -3.427 | | | | | | 0.001** | | | -2.290 | | | 0.022* |  |
| **Left** | -0.04±1 | | -0.53±0.9 | | | 0.06±0.13 | | | -0.04±0.09 |  | | | -2.05 | | | | | | 0.04* | | | -2.394 | | | 0.017* |  |
| **Down** | 0.35±0.6 | | -0.46±0.69 | | | -0.02±0.11 | | | -0.06±0.11 |  | | | 3.427 | | | | | | 0.001** | | | -2.290 | | | 0.022* |  |
| **Gaze-holding**  **Quick-phases (2D vector with both vertical and horizontal components)** | | | | | | | | | | | | | | | | | | | | | | | | | | |
|  | | **Patients** | **Controls** | | ***Z-value*** | | | ***P-value*** | |  | | | |  |  | | | | | |  | | |  | |  |
| **Center**  Frequency  Peak velocity [°/s]  Amplitude [°] | | 0.76±0.55  25.4±13.9  0.9±0.93 | 0.5±0.3  19.2±11.8  0.31±0.2 | | -2.132  -1.276  -2.658 | | | 0.033*  0.028*  0.008* | |  | | | |  |  | | | | | |  | | |  | |  |
| **Left**  Frequency  Peak velocity [°/s]  Amplitude [°] | | 0.64±0.43  36.6±25.5  1.4±1.7 | 0.26±0.16  15.7±33.6  0.52±0.5 | | -2.693  -0.518  -2.012 | | | 0.007*  0.604  0.044* | |  | | | |  |  | | | | | |  | | |  | |  |
| **Right**  Frequency  Peak velocity [°/s]  Amplitude [°] | | 1.04±0.54  21.1±15.1  0.75±0.6 | 0.56±0.3  24.4±19  0.28±0.2 | | -2.406  -0.262  -2.531 | | | 0.016*  0.793  0.011* | |  | | | |  |  | | | | | |  | | |  | |  |
| **Down**  Frequency  Peak velocity [°/s]  Amplitude [°] | | 0.65±0.52  26.5±22.3  1.14±1.38 | 0.45±0.35  36.5±58.5  0.57±0.87 | | -1.124  -0.121  -1.154 | | | 0.261  0.904  0.249 | |  | | | |  |  | | | | | |  | | |  | |  |
| ***Optokinetic nystagmus***  ***Mean slow-phase velocity, [°/s]^d^***  ***Patients Controls*** | | | | | | | | | | | | | | | | | | | | | | | | | | |
|  | ***Horizontal*** | | ***Vertical*** | | ***Horizontal*** | | | ***Vertical*** | | ***Z-value horizontal*** | | | | ***P-value horizontal*** | | | ***Z-value vertical*** | | | | ***P-value vertical*** | | |  | |  |
| **Left** | -1.9±2.4 | | -0.8±1.2 | | -5±1.4 | | | -0.2±0.9 | | -3.801 | | | | <0.0005** | | | -1.462 | | | | 0.144 | | |  | |  |
| **Right** | 3.1±2.4 | | -0.5±1.3 | | 5.1±1.7 | | | -0.2±0.7 | | -2.397 | | | | 0.017* | | | -0.482 | | | | 0.63 | | |  | |  |
| **Up** | -0.3±0.6 | | 2.4±2.5 | | -0.03±0.6 | | | 4.2±1.3 | | -1.286 | | | | 0.198 | | | -2.397 | | | | 0.017* | | |  | |  |
| **Down** | 0.4±0.6 | | -3.4±2 | | 0.2±0.5 | | | -4 ±1.8 | | -0.599 | | | | 0.549 | | | -0.906 | | | | 0.365 | | |  | |  |
| ***Optokinetic nystagmus: quick phases***  ***Mean peak velocity [°/s]*** | | | | | | | | | | | | | | | | | | | | | | | | | | |
| **Left** | 17.6±37.4 | | 8.6±33.7 | 85.7±28.6 | | | | 6.8±23.6 | | -4.503 | | | | <0.0005** | | | -0.322 | | | | 0.748 | | |  | |  |
| **Right** | -17.6±38.9 | | 8.7±39.7 | -84.3±37.1 | | | | -1.96±31.9 | | -3.98 | | | | <0.0005** | | | -0.088 | | | | 0.93 | | |  | |  |
| **Up** | -1.3±40.9 | | -42.8±57.6 | -3.2±16.2 | | | | -68.8±29.7 | | -1.349 | | | | 0.177 | | | -1.525 | | | | 0.127 | | |  | |  |
| **Down** | -9.1±19 | | 73.3±51.8 | -1.6±13.4 | | | | 66.9±38.1 | | -0.878 | | | | 0.38 | | | -0.234 | | | | 0.815 | | |  | |  |
| ***Parameter*** | ***Patient Total*** | |  | ***Controls*** | | | |  | | ***Z-value*** | | | | ***P-value*** | | | ***1. timepoint*** | | | | ***2. timepoint*** | | | ***Z-value*** | | ***P-value*** |
| ***Gain of vestibulo-ocular reflex***  ***Eye velocity/Head velocity*** | | | | | | | | | | | | | | | | | | | | | | | | | | |
| **Gain Right** | 0.69±0.37 | |  | | 1.1±0.14 | | |  | | -3.708 | | | | <0.001** | | 0.7±0.34 | | | | | 0.9±0.37 | | | -1.956 | | 0.05 |
| **Gain Left** | 0.64±0.39 | |  | | 1.08±0.11 | | |  | | -3.868 | | | | <0.0005** | | 0.69±0.38 | | | | | 0.75±0.27 | | | -0.356 | | 0.722 |
| **Gain horizontal** | 0.66±0.37 | |  | | 1.1±0.11 | | |  | | -4.05 | | | | <0.0005** | | 0.69±0.35 | | | | | 0.8±0.30 | | | -1.778 | | 0.075 |

^a^ Due to differences in the sample size smooth pursuit gain of all patients measured twice was analyzed as a group, regardless of age.

^b^ Minus sign indicates the direction of the movement.

^c^ Eye movement drift in both horizontal and vertical planes.

^d^ No longitudinal analysis performed.

* Significance niveau *p* < 0.05

** Significance niveau *p* < 0.001
